# Chlorinated Phospholipids and Fatty Acids: (Patho)physiological Relevance, Potential Toxicity, and Analysis of Lipid Chlorohydrins

**DOI:** 10.1155/2016/8386362

**Published:** 2016-12-20

**Authors:** Jenny Schröter, Jürgen Schiller

**Affiliations:** Institute for Medical Physics and Biophysics, Faculty of Medicine, Leipzig University, Leipzig, Germany

## Abstract

Chlorinated phospholipids are formed by the reaction of hypochlorous acid (HOCl), generated by the enzyme myeloperoxidase under inflammatory conditions, and the unsaturated fatty acyl residues or the head group. In the first case the generated chlorohydrins are both proinflammatory and cytotoxic, thus having a significant impact on the structures of biomembranes. The latter case leads to chloramines, the properties of which are by far less well understood. Since HOCl is also widely used as a disinfecting and antibacterial agent in medicinal, industrial, and domestic applications, it may represent an additional source of danger in the case of abuse or mishandling. This review discusses the reaction behavior of in vivo generated HOCl and biomolecules like DNA, proteins, and carbohydrates but will focus on phospholipids. Not only the beneficial and pathological (toxic) effects of chlorinated lipids but also the importance of these chlorinated species is discussed. Some selected cleavage products of (chlorinated) phospholipids and plasmalogens such as lysophospholipids, (chlorinated) free fatty acids and *α*-chloro fatty aldehydes, which are all well known to massively contribute to inflammatory diseases associated with oxidative stress, will be also discussed. Finally, common analytical methods to study these compounds will be reviewed with focus on mass spectrometric techniques.

## 1. Introduction

Inflammation, as an adaptive response to noxious stimuli such as infection or tissue injury, is an important and complex physiological process mediated by the immune system [1]. In general, a homeostatically controlled inflammatory response is a beneficial “weapon” of the organism to “fight” against infections and infiltrating microorganisms such as bacteria, but a tissue malfunction or homeostatic imbalance in favor of proinflammatory mediators can become very harmful to mammals due to the permanent stimulation of immune cells such as granulocytes or macrophages. A dysregulated immune response may lead, for instance, to tissue degeneration as observed in the loss of cartilage during rheumatoid arthritis [2]. Neutrophilic granulocytes (a crucial cellular part of the immune system) activated during chronic inflammation generate not only proteolytic enzymes and antibacterial proteins but also considerable amounts of reactive oxygen and nitrogen species (ROS, RNS) including both nonradical species (hypochlorous acid (HOCl) and peroxynitrite (ONOO^−^)) and different free radicals, which are, transient compounds with at least one unpaired electron (superoxide anion (O_2_^∙−^), nitric oxide (NO^∙^)) [3]. It is important to note that common atmospheric oxygen (“triplet oxygen”, O_2_) already represents a diradical (^∙^O-O^∙^) where the spins of the valence electrons are in parallel orientation. This ^3^Σg state is rather stable and explains why oxygen undergoes reactions only at elevated temperatures or under enzyme catalysis. This behavior changes significantly if singlet oxygen is considered: in this state (^1^Σg) oxygen is much more reactive due to the antiparallel orientation of the valence electrons. A more detailed discussion of these aspects is, however, beyond the scope of this paper. Although oxygen is needed by all aerobic organisms, its concentration has to be tightly regulated: the replacement of atmospheric air by pure (100%) O_2_ would be strongly toxic for living organisms. Animals used for experimental purposes die after a few days in a pure oxygen atmosphere as a result of excessive oxidation reactions, which cannot be compensated by the organism. It has also been shown that the tumor incidence increases if the oxygen partial pressure in the air exceeds only slightly the standard value of about 21% [4].

### 1.1. Generation of HOCl and Other Reactive Oxygen Species

O_2_ is converted by a number of different enzyme-triggered reactions into even more reactive compounds. In the first step, O_2_ is converted into superoxide (HOO^*∙*^), which exists at physiological pH primarily in the form of the deprotonated superoxide anion radical (O_2_^∙−^). These radicals dismutate either spontaneously or particularly in the presence of the enzyme superoxide dismutase (SOD) into hydrogen peroxide (H_2_O_2_). The enzyme myeloperoxidase (MPO) converts H_2_O_2_ and chloride ions into HOCl, a highly reactive chlorine species (RCS) reacting with the majority of biologically relevant compounds.

HOCl (including its salt sodium hypochlorite (NaOCl)) as a molecular (2-electron) agent has a strong oxidizing and chlorinating ability to ensure high bactericidal and cytotoxic properties. This makes HOCl one of the most powerful in vivo oxidants. Accordingly, HOCl is also used as bleaching, disinfecting, and antiseptic agent for medicinal, industrial, and domestic applications [5]. Normally, the commercially available hypochlorite solution is more stable and much less reactive than the free acid (HOCl). However, both species are (due to the pK value of about 7.53) nearly equally concentrated at physiological pH (7.4). This implies that typical household cleaners (such as “Chlorix”) can be as harmful as the in vivo generated HOCl in the case of abuse or mishandling [6]: damages of virtually all relevant biomolecules as well as many tissues may be induced [7]. Some of these generated so-called disinfection by-products show evidence for their cytotoxic, genotoxic, teratogenic, and carcinogenic potential and this important aspect was recently reviewed [8–10]. Some typical pathological and toxic adverse effects in the case of abuse or mishandling of industrial or medicinal used HOCl are summarized in Table 1 and clearly indicate the sensitivity of health tissues to oxidative stress.

### 1.2. Reactivity of HOCl with Lipids

**Figure 1:**
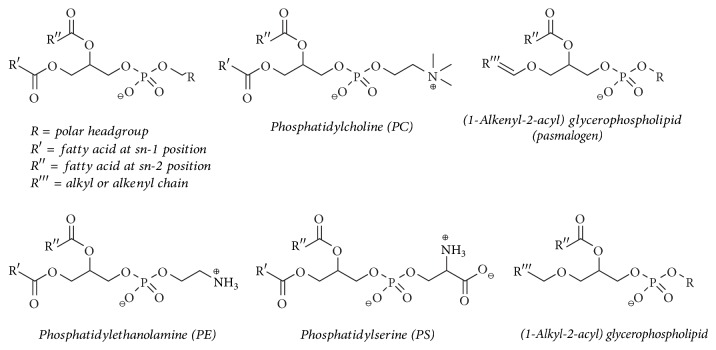
Selected glycerophospholipids (PLs) which will be relevant for this review. Note that phosphatidylcholine (PC) and ethanolamine (PE) are zwitterionic lipids, while PS is an example of a negatively charged PL. PLs in biological samples often contain a saturated fatty acyl residue in* sn*-1 position, while that in* sn*-2 position is normally unsaturated. Ether PL are characterized either by an alkenyl or alkyl linkage in* sn*-1 position at the glycerol backbone. Alkenyl ether lipids are often called “plasmalogens.”

In contrast to free radicals such as hydroxyl radicals (HO^∙^) and nitric oxide (NO^∙^), HOCl is characterized by a more selective reaction behavior. We will discuss the HOCl-induced changes of lipids [11] focusing on the phospholipids (PLs) shown in Figure 1.

The majority of PLs consist of a glycerol backbone, esterified with two varying organic fatty acids and one molecule of phosphoric acid. The resulting phosphatidic acid (PA) is again able, via ester condensation with different alcohols, to form a large variety of PL species like phosphatidylcholine (PC) and phosphatidylethanolamine (PE) as neutral but zwitterionic representatives or phosphatidylserine (PS) as a typical example of a negatively charged PL. There are also ether PLs: the most important ones are the “plasmalogens” (often from PC and PE species) which possess a vinyl ether in the* sn*-1 position and can be formally considered as the reaction products of a fatty aldehyde with a hydroxyl group of the glycerol.

**Figure 2:**
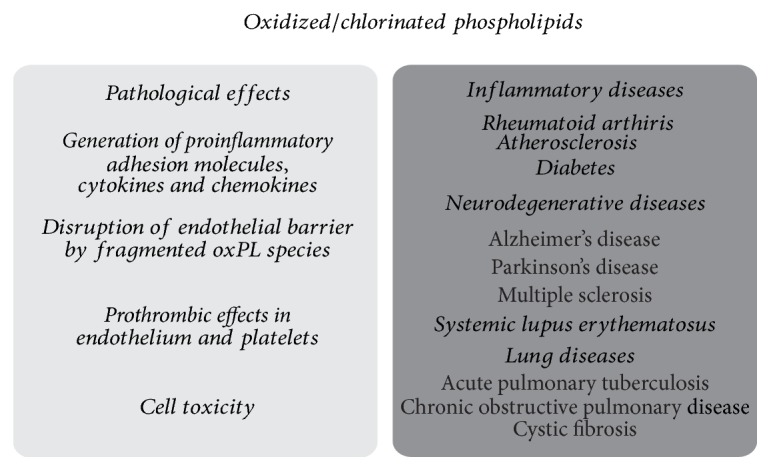
Summary of the known pathological effects mediated by oxidized or halogenated PLs (left) and of the inflammatory diseases in which these lipids are involved (right).

According to the high amount of PLs in biological membranes, they are important targets for peroxidation by free radicals or oxidation/chlorination by enzymatically generated agents. Those PLs, especially, containing highly unsaturated fatty acyl residues (particularly arachidonic acid), vinyl ether linkages, and/or reactive polar head groups (as in PE or PS) are most vulnerable to oxidation processes [12]. It is generally accepted that oxidized/chlorinated PLs exhibit significant effects in acute and chronic inflammation [13] and are thus associated with inflammatory diseases such as rheumatoid arthritis, neurodegenerative diseases, atherosclerosis, diabetes, systemic lupus erythematosus, and lung infections (Figure 2) [7, 14].

Since oxidized PLs exist in a tremendous diversity in biological samples, their detection in crude mixtures is still challenging. To overcome this problem, many different analytical approaches, in particular mass spectrometry (MS) based methods, were developed in the last decades and can now be considered as the most powerful tools in the field of “lipidomics.” “Lipidomics” is a relatively new “omics” approach which focuses on the identification of key species in the lipid metabolism, lipid biomarkers, and lipid signaling alterations in both health and disease [15]. This aspect will be discussed later in this review. However, it has to be emphasized already here that some lipid oxidation products represent transient compounds which either decay into other (more stable) products or react with other biomolecules, which makes the analysis of (at least) some lipid oxidation products a challenging task. Furthermore, the oxidation/chlorination of free fatty acids (FFAs) and especially polyunsaturated fatty acids (PUFAs) by ROS leads also to an enormous pool of bioactive lipid mediators. The most important and intensively investigated classes are the eicosanoid mediators, which are involved in different biological processes such as bone metabolism, nerve development, wound healing, immune responses, and inflammation [16]. However, FFAs represent detergents and are known to destabilize membranes if they are present in elevated amounts [17]. Since they are known as low abundant transient products with a limited stability, the analysis of oxidized/chlorinated FFAs is even more difficult than the investigation of oxidatively modified PLs [16]. Briefly, the search for new lipid and FFA biomarkers containing oxidized moieties in order to diagnose inflammatory processes at an early stage of disease is only realizable with highly specific and particularly sensitive analytical methods which need to be continuously improved.

**Figure 3:**
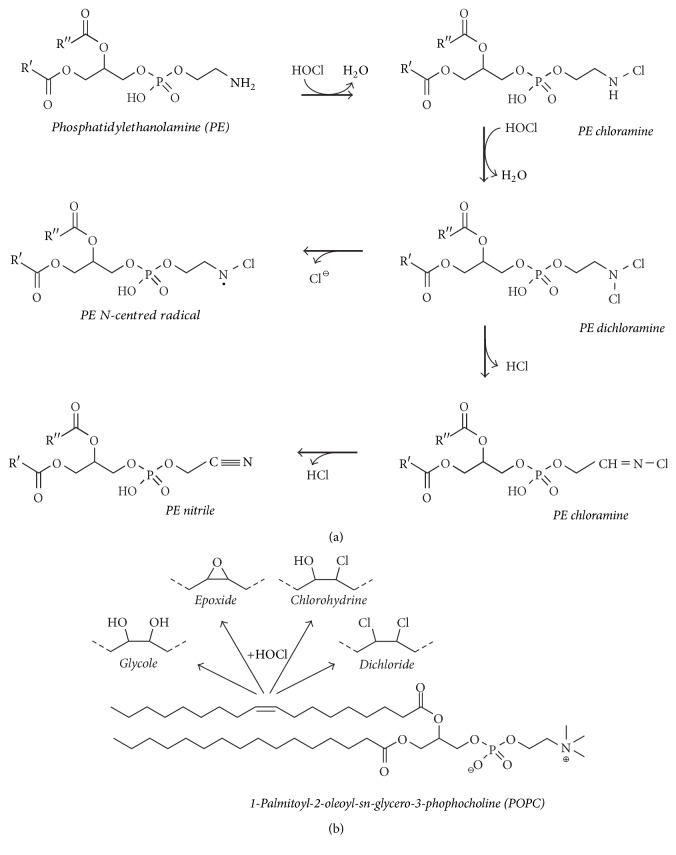
Survey of selected products if an unsaturated PL either with a reactive headgroup (PE, (a)) or with an inert headgroup (PC, (b)) reacts with hypochlorous acid (HOCl). Note the complexity of the reactions and product patterns even if only a single PL is oxidized by HOCl.

In this review, we will focus on the PL and FFA oxidation/chlorination by selected agents with the emphasis on HOCl. Some selected products of PC and PE are illustrated in Figure 3.

We will summarize (i) the potential beneficial properties of HOCl and its chlorinated products (particularly chlorohydrins), (ii) the performed in vitro studies which were particularly aimed to find new biomarkers, and (iii) the predominantly reported negative properties of chlorohydrins and other chlorinated species leading to inflammatory diseases. In the last chapter we will provide an overview of the currently used analytical methods to characterize the oxidized/chlorinated products after PL and FFA treatment with HOCl, focusing on MS methods.

## 2. In Vivo Generation of HOCl by the MPO-H_2_O_2_ System

### 2.1. Release of the MPO-H_2_O_2_ System by Neutrophils

Neutrophilic granulocytes, which are one subtype of the polymorphonuclear leukocytes (PMNs), are known to play a major role during inflammatory processes. Three types of neutrophilic granules are consecutively generated during the maturation process of the cells: primary (azurophilic), secondary (specific), and tertiary (gelatinase) granules that contain the proinflammatory proteins MPO, lactoferrin, and metalloproteinase 9 (which comprises actually different proteins), respectively [18]. In healthy individuals the release of neutrophils from bone marrow is tightly regulated and known to eliminate pathogens either intra- or extracellularly via phagocytosis or the release of neutrophil extracellular traps (NETs) [19].

Since the total MPO concentration is about 5% of the whole neutrophil proteins [20], it is one of the major physiologically occurring components in these phagocytes and even increases massively during inflammatory processes. Thus, it is a widely studied enzyme in the fields of inflammation as well as oxidative stress and is often used as a biomarker for many diseases including atherosclerosis, rheumatoid arthritis, and neurodegenerative diseases [21]. The most common diseases linked with the MPO system are summarized in Table 2 and include the changes of the MPO concentration or activity, the investigated matrix, and finally the method by which the MPO amount and/or its activity was determined. For further details see the references provided in Table 2.

During chronic infectious and inflammatory diseases the increased numbers of neutrophils permanently release not only MPO but also ROS during the “respiratory burst” that is triggered by the enzyme NADPH oxidase [22]. This may result, particularly in chronic inflammation, in both immunopathological processes and secondary damages in living organisms [23].

**Figure 4:**
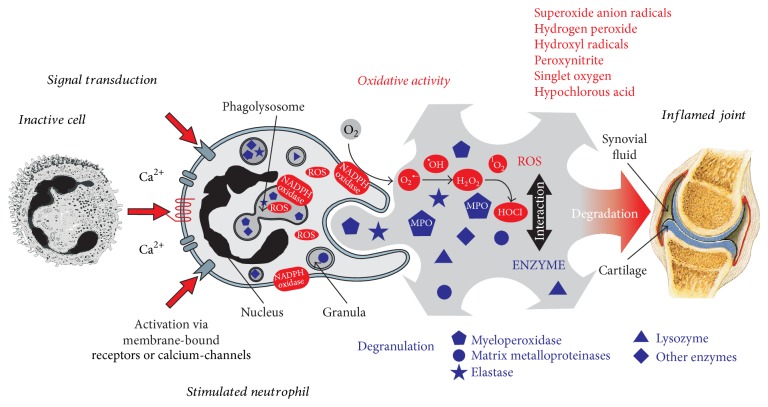
Overview of the released reactive oxygen species (ROS) and antimicrobial enzymes by an activated neutrophil during the inflammatory process in an inflamed joint. Due to the release of highly toxic ROS, most of them (including HOCl) do not discriminate between microbial and “healthy” targets, leading to an uncontrolled oxidation of all biomolecules and a persistent inflammation (reprinted from Schiller et al. [25], with permission from Elsevier).

Since rheumatoid arthritis is a widespread disease with an annual incidence of 25–50/100 000 and a prevalence of 0.5–1.0% in North America and Europe [24] and has, even more, a high socioeconomic weight, it is mandatory to find early stage biomarkers to enable early treatment of the disease. The evaluation of the processes of cartilage degradation during inflammatory joint diseases is also a major research topic in our laboratory.  Figure 4 shows, exemplarily for all inflammatory processes, the release of ROS (HOCl, H_2_O_2_, O_2_^∙−^) as well as antimicrobial enzymes (e.g., MPO, elastase, lysozyme) by an activated neutrophil that infiltrated into the joint space during acute joint inflammation [25]. Symptoms of rheumatoid arthritis may have periods of remission in which the symptoms disappear, alternating with periods of flare-ups, which is correlated with the extent of inflammation.

These cartilage damaging agents do not discriminate between microbial and host target molecules and will thus oxidize a large number of biologically healthy targets such as cells, proteins, or polysaccharides leading to a persistent inflammation. For a detailed description of neutrophilic activity in immune response and inflammation see also [19, 26, 27]. Furthermore, a detailed review about the physiological properties of ROS and their regulatory enzymes has recently been published by Zuo and coworkers [28].

### 2.2. HOCl Generation during the “Chlorination” Cycle

**Figure 5:**
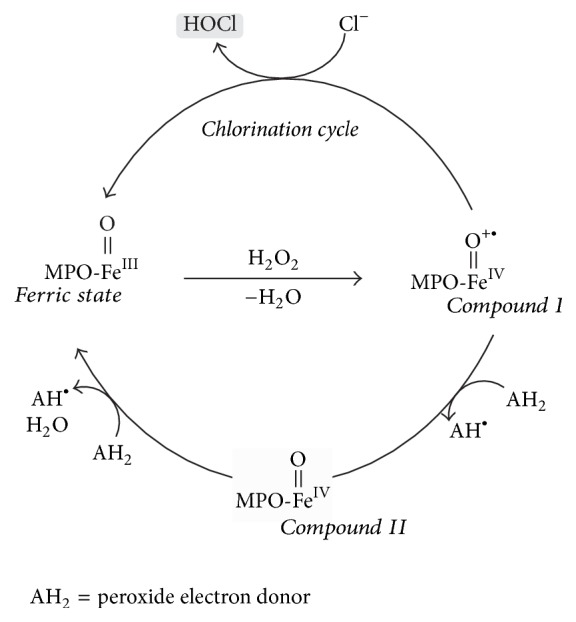
Generation of HOCl by MPO via a chlorination cycle. In the first step, MPO is oxidized by hydrogen peroxide (H_2_O_2_) into the short-lived compound I that contains a Fe^IV^ radical cation center. Compound I is further regenerated to the educt, the ferric state MPO, either by two consecutive 1-electron steps via compound II or by oxidizing Cl^−^ to HOCl (modified in accordance with [33]).

As a member of the heme peroxidase family in myeloid cells, MPO becomes oxidized at the Fe^III^-ion position by H_2_O_2_ under the formation of a short-lived intermediate,* compound I*, with a Fe^IV^ radical cation center [29, 30]. Afterwards,* compound I* is regenerated to the ferric state (three-valent) of MPO, either in two consecutive 1-electron steps (via* compound II*) or by oxidizing Cl^−^ to HOCl (second order rate constant: (2.5 ± 0.3) × 10^4^ M^−1^s^−1^ [31]) during the chlorination cycle (Figure 5). Further details are available in a detailed review by Arnhold and Flemmig [32].

It is nearly impossible to determine the HOCl concentration under in vivo conditions, as HOCl is continuously generated by MPO (at least if H_2_O_2_ and Cl^−^ are present in sufficient amounts) but instantaneously consumed by its reaction with potential target molecules. Therefore, the stationary HOCl concentration is close to zero. However, there are also some papers available which provide dedicated HOCl amounts. Since HOCl is known from these reports to be highly abundant under inflammatory conditions (50–100 mM) [34] and even under physiological conditions (0.34 mM) in the extracellular space [35], it was assumed for many years that it is the only physiologically relevant halogenated product of MPO [36]. Nowadays, it is well accepted that MPO generates, even under physiological conditions, also hypobromous acid (HOBr) and hypothiocyanite (HOSCN), even if the corresponding bromide (20–100 *μ*M) and thiocyanate (20–120 *μ*M) concentrations are much lower than the chloride (100–140 *μ*M) plasma concentrations [31]. Moreover, even cyanate/isocyanate, which are particularly important in human saliva [37], can be generated by MPO, however, to a minor extent [6, 38].

For example, Senthilmohan and Kettle [6] suggested HOBr could be an in vivo relevant major product of MPO at physiological conditions (second order rate constant: (1.1 ± 0.1) × 10^6^ M^−1^ s^−1^ [31]) and should be considered if investigations of inflammatory processes are performed. This is surprising, because Br^−^ is about three orders of magnitude less abundant in comparison to Cl^−^ (about 10^−1^ versus 10^−4^ M) and implies that bromide is preferentially metabolized by MPO. Furthermore, HOBr can be generated indirectly by the reaction between HOCl and bromide [39] which is also used to synthesize HOBr in the laboratory:

(1) $$\begin{array}{c} {Br}^{-}+HOCl⟶HOBr+{Cl}^{-} \end{array}$$

Both HOCl and HOBr are strongly oxidizing agents with the ability to modify amino moieties in proteins, DNA or PLs (such as PE and PS) resulting in the generation of chloramines and bromamines as primary products [21].

Although a detailed discussion of these aspects is beyond the scope of our review, there are nowadays increasing indications that HOSCN (in particular compared to HOCl) may have protective effects in the organism: HOSCN shows a marked preference for thiol residues, forming products (particularly long-lived sulfenyl thiocyanate (RS-SCN)) that can be “repaired” by the organism [40], resulting in reversible cellular damage. Since HOCl (or HOBr) may react with SCN^−^ to generate HOSCN (second order rate constant: (9.6 ± 0.5) × 10^4^ M^−1^ s^−1^) [41] this reaction (2) may represent an important way of how the organism gets rid of excessive HOCl (HOBr)

(2) $$\begin{array}{c} {SCN}^{-}+HOCl⟶HOSCN+{Cl}^{-} \end{array}$$

There is still significant interest in HOCl, since it is a specific product of MPO, whereas HOBr is additionally (or even preferentially) produced by the enzyme eosinophilic peroxidase (EPO) [11]. Due to its high toxicity, HOCl released by neutrophils into the phagosome seems to have a high impact in fighting against microbial pathogens and controlling the bacterial population in the host [42]. However, the impaired homeostasis between oxidants and antioxidants, in favor of ROS production, leads to an uncontrolled generation of HOCl, which is associated with chronic inflammatory diseases (Figure 2), may have carcinogenic and mutagenic potential [43], and is even known as a stable but highly reactive neurotoxic oxidant in the brain, where HOCl reacts rapidly with H_2_O_2_, O_2_^∙−^ or nitrite generating ^1^O_2_, HO^∙^, or nitryl chloride (NO_2_Cl) that may contribute to extensive tissue damage [44]. Due to its fast homeostatic consumption, in vivo generated HOCl cannot be measured directly, but either as stable scavenger products or via the determination of the MPO activity in the corresponding body fluid, for instance, by using the well-known conversion of guaiacol into tetrahydroguaiacol [45]. Therefore, different methods were established to measure stable HOCl-induced products, often by absorbance or luminescence methods, but increasingly also by MS [46]. The optimum biomarker for HOCl should be specific, stable, and sensitive to determine [47]. However, due to the fast turnover of HOCl, it is still a challenge to find a biomarker that combines all these properties.

### 2.3. Survey of MPO Activity by HOCl Determination

Although HOCl is known to chlorinate a variety of different biomolecules, amino acids are the most susceptible targets for chlorination by HOCl. The concentration of 3-chlorotyrosine (and to a minor extent 3,5-dichlorotyrosine) is often considered as the biomarker of choice for the MPO activity in vivo by different MS methods [48], because it is highly specific, comparably stable, and its concentration correlates with the MPO activity [49–51]. Nevertheless, 3-chlorotyrosine is, first, only a minor product of the protein chlorination by HOCl and, second, it is only an intermediate in biological systems [52]. Since 3-chlorotyrosine reflects only a small percentage of the absolutely released HOCl, its function as a specific MPO activity biomarker is assumed to be limited [53].

**Figure 6:**
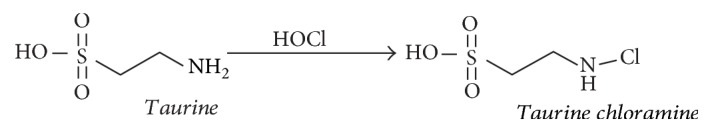
Reaction of taurine, a very important organic (sulfonic) acid which is highly abundant in neutrophils, and HOCl leading to the comparably stable oxidation/chlorination product taurine chloramine, which is used as a marker for the HOCl production rate by MPO and can be easily determined by the taurine chloramine assay. For further details about the assay, see [47].

The second well studied component in MPO activity studies is taurine (2-aminoethanesulfonic acid), because it is highly abundant in neutrophils with a concentration of about 22 mM [54]. Combined with HOCl, taurine serves as a system to study its fast chlorination by hypochlorite, leading to the stable oxidation product taurine chloramine (Figure 6) that can be conveniently measured via enzyme-linked immunosorbent assay (ELISA) [55]. Taurine chloramine is of low toxicity and reasonable stability and possesses both anti-inflammatory and antimicrobial properties. Its role in acute inflammatory processes is not yet fully understood but has been comprehensively discussed by Marcinkiewicz and Kontny [56].

Smaller amounts of HOCl can be determined by using dyes such as 5-thio-2-nitrobenzoic acid (TNB) and 3,3′,5,5′-tetramethylbenzidine (TMB). Using TMB, taurine chloramine is treated with iodide leading to the formation of ICl which is instantly hydrolyzed to hypoiodous acid (HOI). TMB may be used afterwards directly as the chromophore, because it is readily oxidized by HOI to a strongly absorbing blue product [57] that allows the determination of *μ*M quantities of HOCl.

In addition to the TNB and TMB assays, the NAD(P)H assay, the ascorbate assay, and the hydrogen peroxide consumption can also be used to determine the HOCl generation by MPO. These methods were reviewed by Kettle et al. only recently in 2014 [47]. Other potential biomarkers for the MPO/HOCl chlorinating system are either plasmalogens (which will be discussed in more detail later) or glutathione (*γ*-L-glutamyl-L-cysteinyl-glycin), which result in unsaturated (lyso)PLs and *α*-chloro aldehydes or glutathione sulfonamide, respectively. These compounds are easily detectable by MS methods which will be shortly discussed at the end of this paper [58, 59].

## 3. Reactivity of In Vivo Generated HOCl

Due to the fact that the generated HOCl is directly consumed by its reaction with abundant biomolecules, there is increasing evidence that the distribution of HOCl is not homogeneous: MPO is a strongly cationic (positively charged) protein and will therefore be particularly located close to negatively charged compounds, either negatively charged proteins and carbohydrates such as heparin [60], or negatively charged PLs, particularly PS [61], which is almost completely located in the inner leaflet of a membrane, at least in living, nonapoptotic cells. This suggests that MPO binds to apoptotic cells, which leads to an inhomogeneous distribution of HOCl within tissues.

### 3.1. Generation of Biomolecular Radicals

As it is produced in large quantities and represents a reactive two-electron oxidizing agent in human beings under inflammatory conditions, HOCl is several orders of magnitude more reactive than other oxidants like H_2_O_2_, (lipid) hydroperoxides, or peroxynitrite [62]. High reactivity normally goes along with nonselectivity. Therefore, HOCl affects not only infiltrated microorganisms, but also “normal” intracellular enzymes, nucleotides, carbohydrates, or lipids in the body with the capacity to change membrane compositions [63]. Nevertheless, HOCl is also a specific oxidizing/halogenating agent that discriminates between different functional groups and reacts normally in the following order: reduced sulfur moieties (thiols and thioethers) > primary and secondary amines > phenols, tertiary amines > double bonds, other aromatics, carbonyls and amides [64]. A survey of calculated and/or experimentally determined second order rate constants (*k*) are available in [65].

**Figure 7:**
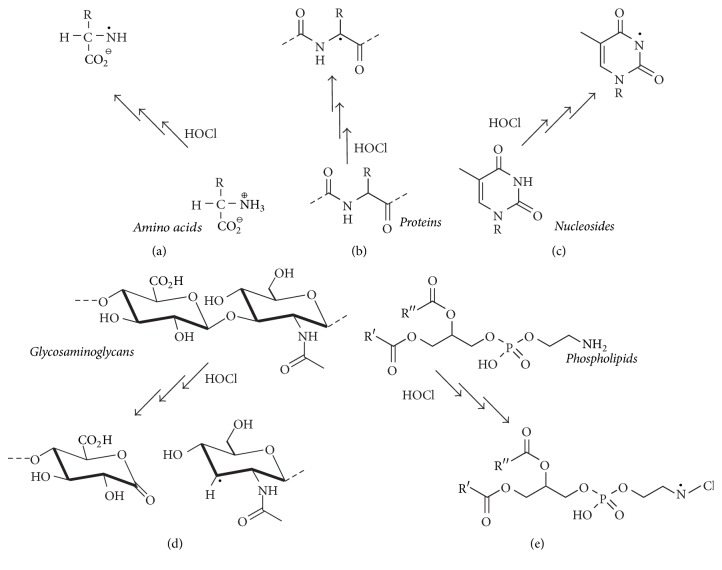
Overview of generated radical species by the reaction of HOCl and different biomolecules. The HOCl-induced chlorination of free amino acids (a, [66]), proteins (b, [66]), nucleosides (c, [67]), glycosaminoglycans (d, [68]), and PLs (e, [69]) leads in various consecutive reactions (implied by three arrows) to different radical species which may cause oxidative stress and favor the development of many diseases.

HOCl has the ability to generate free radicals and stable cleavage products by its reaction with small inorganic molecules or functional groups of biomolecules such as proteins, lipids, nucleotides, and carbohydrates.  Figure 7 shows some selected examples for the generation of radicals from biomolecules after treatment with HOCl.

#### 3.1.1. Amino Acids and Proteins

Free amino acids can be affected by HOCl in a concentration-dependent reaction at either the sulfur or the amino group in the side chain or the *α*-amino group of amino acids, the reaction at the sulfhydryl group being the preferred one: previous investigations provided evidence that the amino groups are not affected at all until all sulfhydryl residues have reacted [70]. The chlorination by HOCl at the *α*-amino group results in transient amino acid chloramines (AACLs), which are known to induce further reactions more selectively than free HOCl. Therefore, AACLs are potential secondary mediators of HOCl-induced injuries [71]. As the most abundant free amino acid (however, not in the strict classical sense, as -COOH is replaced by -SO_3_H in taurine), taurine chloramine (Figure 6) is one of the most important products of amino acid chlorination in human PMNs and was already shortly described in the chapter before. In general, AACLs seem to affect biological targets such as cytoplasmic molecules or glutathione and mediate the HOCl-related toxicity leading to apoptotic cell death [72]. Even without any further oxidative reactions, *α*-amino group AACLs decompose in the presence of water under formation of the corresponding aldehydes [50] or generate, via a one electron pathway, also* N*-centered radicals (Figure 7(a)), which are quickly converted into* C*-centered radicals by different mechanisms, for instance, inter- or intramolecular transport of a proton or decarboxylation [66]. These AACLs decompose to free radicals, which are known to be cytotoxic for tissues and organs [33] and the organism has to try to keep their concentration limited by increased degradation and/or the uptake of antioxidants. It was also shown that HOCl reacts with the same functional groups in proteins as in the case of free amino acids, but these reactions are normally somewhat slower, presumably due to the increased viscosity of protein solutions [73].

Due to their high physiological concentrations in cells, plasma, and most tissues, proteins are actually the most relevant targets for in vivo modification by HOCl and other oxidants causing numerous posttranslational modifications such as the oxidation of sulfhydryl groups and the chlorination of aromatic side chains or the chlorination of amino groups in the side chains and some other side reactions [42]. It is assumed that HOCl primarily affects more readily the functional groups in the side chains of proteins than in the peptide linkages [74]. The side chain chlorination comprises aggregation, oligomerization, destabilization, fragmentation, and/or degradation steps [42] after the formation of an* N*-centered radical which is further converted to a* C*-centered radical (Figure 7(b)) often catalyzed by transition metal ions (e.g., Cu^+^) [66]. Cysteine and methionine (sulfur-containing amino acids) are characterized by an about 100-fold higher reactivity with HOCl than any other functional group of proteins or other biomolecules. The HOCl-initiated chlorination of cysteine leads to labile sulfenyl chloride (R-S-Cl) and sulfenic acid (R-S-OH) intermediates, which are both known to undergo further oxidation steps with HOCl [75]. These irreversible modifications result in sulfinic (R-SO_2_H) or sulfonic (R-SO_3_H) acids as well as thiosulfinate products (R-S-SO-R_2_), the formation of which is accompanied by protein decomposition [5]. Methionine is almost as readily affected as cysteine and may either generate (under in vivo conditions) the reversible methionine sulfoxide or the irreversible dehydromethionine [76]. Beside sulfur-containing amino acids, the amino groups in lysine, histidine, and arginine are also preferred targets for HOCl. Although the reaction rates are reduced in comparison to cysteine, the majority of reaction products are generated irreversibly in vivo and are thus prone to further reactions with other potential reactive protein sites and/or other biomolecules [74].

#### 3.1.2. Nucleic Acids and DNA

The HOCl-induced oxidation of nucleic acids in a DNA strand normally affects the nitrogenous base either at the primary nitrogen (“exocyclic”) in adenosine, guanosine, or cytosine or at the secondary nitrogen (“endocyclic”) in guanosine, uridine, or thymidine, the reaction at the endocyclic nitrogen seemingly being preferred [11]. These* N*-chlorinated products are intermediates and subsequently converted into more stable chlorinated products such as 5-chlorocytosine, 5-chlorouracil, 5-chloro-(2′-deoxy)-cytidine, 8-chloroadenine, 8-chloro-(2′-deoxy)adenosine, and 8-chloro-(2′-deoxy)guanosine [77, 78]. Using electron paramagnetic resonance (EPR) in combination with spin trapping to enhance the lifetime of the radicals of interest, the generation of* N*-centered radicals of nucleosides (Figure 7(c)) from transient chloramines could be monitored by disrupting the double-stranded DNA [67]. The tendency of radical formation of isolated nucleosides as well as polynucleotides can be sorted as follows: cytosine > adenosine > guanosine > thymidine [67, 79]. Some of these products were determined both in inflammation-related cell cultures and in inflamed tissues and are known to cause the dissociation of the double-stranded DNA (cleavage of the hydrogen bonds) and are therefore potential biomarkers of early inflammatory processes [80]. It is known that the concentrations of chlorinated nucleosides (5-chlorouracil) are elevated in the inflammatory exudate of humans and rats as well as in atherosclerotic plaques (AP) [55]. Moreover, there is evidence for the cytotoxic and mutagenic potential of chlorinated nucleic acids or chlorinated DNA moieties (e.g., [77, 81–84]). These cytotoxic effects (i.e., frameshift mutations or base pair substitutions) can be caused by both lipid peroxidation products and HOCl-mediated protein-DNA crosslinks. Under neuropathological conditions, HOCl is continuously released from PMNs or microglia to diffuse into the brain parenchyma and maintains chlorinative stress resulting in neurodegeneration [44]. Even if noncovalent ionic interactions of DNA and proteins are physiologically very important for normal cellular functions [85], the oxidation of covalently bound protein-DNA cross linkages leads to inadequate DNA repair [86] and to mutagenic and cytotoxic effects of phagocytes on microbial pathogens and host tissue [87].

#### 3.1.3. Carbohydrates

Oxidized carbohydrates were investigated to a much lesser extent, presumably due to their complexity [88]. As already discussed in the context of other biomolecules, free amino groups in sugars such as glucosamine are the preferred targets for HOCl. Although the reaction between HOCl and common monosaccharides is comparably slow [89, 90], the HOCl-induced transformation of free amino groups in sugars was reported to be even faster than the modification of the* N*-terminal amino group of peptides [91]. Moreover, HOCl causes the generation of chloramides (R-NCl-C(O)-R_2_) from amide groups (R-NH-C(O)-R_2_) of glycosaminoglycans (GAGs) in the extracellular matrix [92]. Since hyaluronan and chondroitin sulfate regulate different cellular and tissue functions and possess an* N*-acetyl group in the repeating disaccharide unit, they are the most intensely investigated GAGs in the extracellular matrix [93], another reason why GAGs are intensively studied is their abundance in cartilage (chondroitin sulfate) or synovial fluid (hyaluronan), where inflammation plays a major role [88]. Due to the poor oxidizing properties of the initially generated chloramides, they are prone to reacting with different reducing agents including radicals [92] and are thus only transient products. Chloramides decompose to* N*-centered radicals (R-N^∙^-C(O)-R_2_) followed by* C*-centered radicals, leading finally to the site-specific fragmentation of the glycosidic linkages in the polysaccharide chain (Figure 7(d)) under generation of GAG oligosaccharides [68]. These reactions are stimulated by the presence of low-valent transition metals such as Fe^2+^ or Cu^+^ via a one-electron reduction of the chloramide group [68]. Since GAGs are known to be involved in important cellular functions, it is assumed that oxidatively modified GAGs may lead to altered cell migration, adhesion, proliferation, growth, and even phenotyping [33]. Finally, it is known that high mass hyaluronan has anti-inflammatory properties, while low mass hyaluronan is proinflammatory [94]. Therefore, changes of the molecular weight of hyaluronan are of major importance.

#### 3.1.4. Phospholipids

The last important class of biomolecules that can be affected by HOCl is represented by lipids, particularly PLs. It is well known that HOCl reacts with either the double bonds of the acyl chains of unsaturated PLs or the reactive functional groups in the headgroup (e.g., the amino residues in PE and PS). Although the focus of this review is on the addition of HOCl as a nonradical (molecular) agent to the double bonds of PLs which leads to chlorohydrin generation, HOCl can also trigger the formation of* N*-centered radicals in PE and PS by reaction with the nitrogen in the headgroup. The first step of the amino modification by HOCl is the generation of monochloramine, which can be converted (in the presence of a sufficient excess of HOCl) into the corresponding dichloramine [95], which are both only fairly stable compounds. Using EPR with spin trapping, Kawai et al. could monitor the generation of an* N*-centered radical from the PE dichloramine (Figure 7(e)) [69]. Via direct or radical-mediated processes, the elimination of HCl may also occur and result in the generation of the corresponding nitrile (Equation (3)), while an aldehyde is the final product of the hydrolysis of the monochloramine (vide supra)

(3) $$\begin{array}{c} R\text{-}{CH}_{2}\text{-}N{Cl}_{2}⟶R\text{-}CN+2HCl \end{array}$$

We will not focus on these aspects here to a major extent. The reader who is particularly interested in the related reaction mechanisms and further details about free radical formation from biologically important molecules by HOCl is referred to the excellent review by Panasenko and coworkers [33]. However, it is widely accepted that the radicals derived from chloramines may induce or mediate (through, e.g., tocopherol) oxidative modifications of lipids [96].

## 4. Phospholipid Oxidation by HOCl

**Figure 8:**
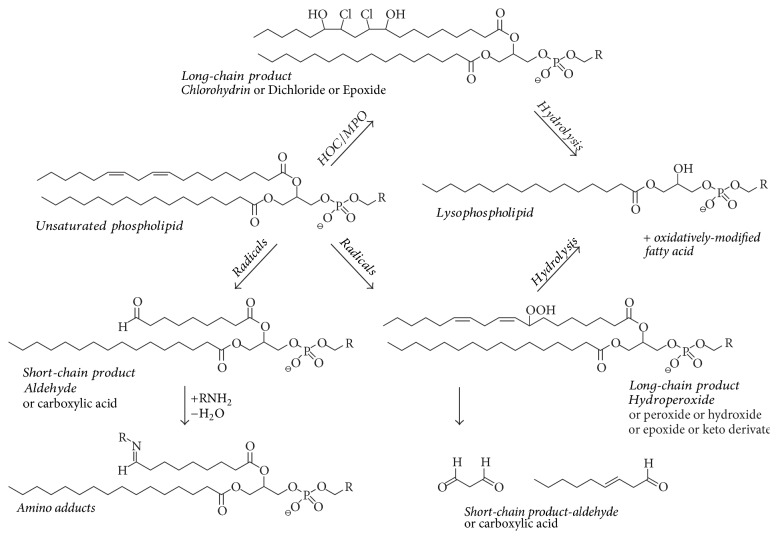
Scheme of potential reaction products from an unsaturated PL under inflammatory conditions in vivo. Please note that the term “long-chain” products refers to compounds that have a higher mass in comparison to the native PL while “short-chain” products have a smaller mass. A similar scheme has been suggested recently [97]. Although lysophospholipids (LPLs) might be also considered as “short-chain” products, they are discussed here as a distinct lipid class. This scheme should be regarded as a very crude survey and not all compounds that play a physiological role are actually listed (reprinted from [98] with permission from Elsevier).

A crude survey of products which may be expected if PLs are subjected to HOCl oxidation is shown in Figure 8 [97].

The most relevant PL oxidation products can be sorted into (i) full chain length products (hydroperoxides, hydroxides, and epoxides), (ii) full-chain rearranged products (isoprostanes and isofurans), and (iii) truncated products (alkanals, alkenals, and hydroxyalkenals) [99]. Although compound class (iii) is currently most intensely investigated, because aldehydes may react (under the particular generation of Schiff bases and other products) with proteins and modulate in this way the activity of enzymes [100], the compounds we will discuss here in more detail belong to group (i): no truncation of the fatty acyl residue occurs if HOCl reacts with the double bonds of PLs or FFAs under generation of chlorohydrins as addition products.

The reaction between olefinic compounds (such as unsaturated lipids) and HOCl is known for more than a hundred years, including discussions about which compounds can be considered as the stable final products: chlorohydrins, glycols [101], or epoxides [102]. Nowadays, it is commonly accepted that chlorohydrins (the addition products of HOCl to the double bond) are the most abundant products when lipids are treated with HOCl, even if there are also recent reports that dimeric products of oleic acid may be also generated [103].

Beside PLs (as shown in Figure 8) containing two fatty acyl residues, which are linked to the glycerol by ester linkages, alkenyl-acyl PLs, commonly termed “plasmalogens,” are a second class of PLs that occur in significant amounts in many biological samples, for instance, in neutrophils, macrophages, glia cells, neurons, smooth muscle cells, endothelial cells, cardiac myocytes, and particularly spermatozoa [104, 105]. These plasmalogens are generated by the reaction between glycerol and a fatty aldehyde followed by the elimination of one water molecule and are often characterized by a choline or (even more abundant) an ethanolamine headgroup [106]. Although brain and spermatozoa lipids contain considerable amounts of highly unsaturated fatty acyl residues (such as docosahexaenoic acid, 22 : 6) [107], it is nowadays commonly accepted that the alkenyl ether linkage represents a site of high reactivity with a large variety of ROS [108], much higher in comparison to the “normal” double bonds along a common fatty acyl chain [109]. Therefore, plasmalogens are often considered to represent important biological antioxidants [110]. It is important to note that the resulting plasmalogen LPCs possess an unsaturated fatty acyl residue in the* sn*-1 position [111], which is a remarkable difference in comparison to diacyl-PCs that possess the unsaturated fatty acyl residue normally in the* sn*-2 position and yields exclusively saturated LPCs upon oxidation with HOCl. Therefore, the detection of unsaturated lysophospholipids (LPLs) is normally a clear indication of the presence of plasmalogens [112].

## 5. Beneficial Effects of Lipid Chlorohydrins and Peroxidized Lipids

The majority of authors indicate that lipid oxidation processes and the related products exert a negative effect on the organism and that everyone should attempt to minimize the extent of lipid oxidation, for instance, by the ingestion of antioxidative compounds such as vitamins [113]. However, there are also a few indications that lipid oxidation positively affects the organism [13].

There are indications that oxidized lipids are faster digested by phospholipases (particularly phospholipase A_2_ (PLA_2_), which cleaves exclusively the fatty acyl residue in* sn*-2 position and plays a more prominent role than PLA_1_) than their native (nonoxidized) counterparts. This emphasizes that the organism recognizes oxidized lipids as “foreign” and is thus interested to get rid of these molecules as fast as possible. This increasingly important aspect has been reviewed recently [114]. Conversely, this means that PLA_2_ enzymes (there are many different species) affect oxidized PL more readily: for instance, it could be shown that AP are characterized by an elevated content of lysophosphatidylcholine (LPC), which is presumably generated by the action of PLA_2_ on PC [115]. Although there are many indications that peroxidized lipids are preferentially digested by PLA_2_, the detailed mechanisms behind this finding are not yet completely clear. The two potential reasons are, first, the activity of the enzyme might be actually elevated in the presence of a different substrate, because this substrate fits more readily into the active site of the enzyme, and second, the presence of oxidized lipids may also be accompanied by alterations of the lipid membrane structure and this might lead to improved digestibility [116].

Different effects in comparison to peroxidized lipids have been described for HOCl-modified lipids: halogenated PLs are inhibitory to the enzyme and elevated concentrations of chlorinated (and brominated) PC molecules decrease the activity of secretory PLA_2_ by about 50% [34]. This could be confirmed recently by using bee venom PLA_2_ and liposomes of defined lipid compositions [148]. These notable differences clearly emphasize that more detailed studies of these aspects are necessary.

In general, it is known that oxidized lipids (especially oxidized arachidonyl residues) can induce protective effects by the upregulation of antioxidant genes, inhibition of inflammatory signaling pathways through Nrf2 (nuclear factor erythroid 2-related factor 2) mechanisms [149], antagonism of Toll-like receptors [150], immunomodulating and immunosuppressive action of oxidized PLs in adaptive immunity and autoimmune disease [13], activation of PPARs (peroxisome proliferator-activated receptors) which are known for their anti-inflammatory action, and the protective action against lung edema in acute lung inflammation [151]. As implied before, plasmalogens are also known as antioxidants.

## 6. In Vitro Generation of Chlorohydrins (Cell and Tissue Studies)

Although many papers focus on the effects of (per)oxidized lipids on selected cell lines, the effects of chlorohydrins were studied to a much lesser extent. This might be due to the fact that PL chlorohydrins (unlike, e.g., “oxPAPC” [152]) are nowadays not commercially available. However, this purchasable “oxPAPC,” which is obtained by air oxidation of PAPC, is a relatively crude mixture and contains at least three different substance classes (hydroperoxides, aldehydes, and carboxylic acids). This makes detailed assignments about which compound is responsible for the observed effects very difficult.

In one of the first chlorohydrin-related studies, Carr and coworkers treated human erythrocytes with HOCl [153] and were able to detect the generation of chlorohydrins by means of thin-layer chromatography (TLC) and by using an ELISA assay against chlorohydrins. Since lipid chlorohydrins are more polar and bulky than their parent lipids [154], their impact on membrane stability and integrity was also investigated by measuring the release of hemoglobin from the cells. Different chlorohydrins caused different effects: the addition of HOCl-treated oleic acid to the red blood cells resulted in a rapid (concentration-dependent) lysis of the cells while the effect of cholesterol CH was less pronounced and was also dependent on the type of the CH isomer with the chlorohydrin-3 being the most deleterious one. Further analysis by Vissers and coworkers [155] examining HOCl-lysed red cell ghosts by electron microscopy demonstrated considerable disruption of the cell membrane and provided evidence for complete rupture at an HOCl concentration of about 5 mM.

The same authors [156] extended their work a few years later to another cell system: when human umbilical vein endothelial cells were treated with preformed halohydrins of oleic acid, cell detachment and necrotic death can be induced with increasing doses of the halohydrins, whereas the cells were unaffected by equivalent doses of pure oleic acid as the control. Bromohydrins (which can be obtained by the treatment of lipids with HOBr which can be conveniently generated by mixing HOCl with a small excess of NaBr) caused even more lysis than the corresponding chlorohydrins at equivalent doses, presumably because bromohydrins are more readily incorporated into the endothelial cells. It was also suggested that membrane protein modification is the reaction which is primarily responsible for HOCl-mediated cell lysis [157]. Therefore, further studies of these aspects are required.

It is important to note that chlorohydrins seem to have not only structural relevance but are also actively involved in metabolic pathways. It could be shown [158] that the cellular adenosine triphosphate (ATP) level is significantly diminished when cultured myeloid (HL60) cells are incubated for 24 h with selected chlorohydrins. These effects were additionally compared with the effects of different small aldehydes (such as 4-hydroxy-2-nonenal, HNE) which are known to be generated under inflammatory conditions by cleavage at the double bond position of unsaturated fatty acyl residues. The ATP depletion by the PL chlorohydrins was slightly less than that of HNE, but greater than that of hexanal and trans-2-nonenal, which are all well-known oxidation products of lipids. This indicates that chlorohydrins directly affect the activities of enzymes.

Robaszkiewicz and coworkers [159] studied the effects of chlorohydrin PCs on human erythrocytes. It could be shown that the biophysical properties of PL bilayers of known compositions are altered in the presence of PC chlorohydrins. In particular, changes in erythrocyte shape (echinocyte formation) and aggregation were significantly altered when vesicles containing PC-CH were mixed with the cells. Similar effects were also found [160] when HUVEC-ST (endothelial cells) were treated with PC chlorohydrins. Under these conditions there was a decrease of the mitochondrial potential and an increase in the number of apoptotic cells. These effects were accompanied by an increase in the level of active caspase-3 and caspase-7 and a decrease in glutathione content and the overall antioxidant capacity of the cells. Similar effects were observed in the case of lung epithelial cells and HOCl was found to affect the redox state of A549 cells by oxidation of GSH, inactivation of antioxidant enzymes, and the increase of ROS generation [161].

It could also be shown that induced acute pancreatitis results in a substantial release not only of FFAs but also of the chlorohydrins of both oleic and linoleic acid from adipose tissue while accompanying plasma investigations evidenced only the chlorohydrin of oleic acid. Administration of 250 *μ*M lipid chlorohydrins, the concentration found in ascitic fluid, induces the expression of TNF-*α* and IL-1*β* in peritoneal macrophages and increases the systemic inflammatory response in pancreatitis. Finally, increased concentrations of oleic acid chlorohydrin have been found in the plasma of human patients with pancreatitis [162].

## 7. Chlorohydrins in Diseases

The last example indicates that there is increasing evidence that products derived from the MPO/H_2_O_2_/Cl^−^ system play a crucial role in many diseases and it can be suspected that this neutrophil-derived system is, beside inflammation mediated by T-cells, a major contributor in inflammatory diseases [163, 164]. A survey of different diseases mediated by MPO activity is given in Table 2, showing only the most important diseases due to the limited space available.

### 7.1. Atherosclerosis

There is increasing evidence that many diseases are accompanied by inflammation. This does not only apply for classical inflammatory diseases such as gastritis and arthritis, but also for diseases such as obesity and cancer [165]. The most important and therefore most intensively studied disease is atherosclerosis [166], which is characterized by the thickening, hardening, and loss of elasticity of the walls of arteries up to the buildup of fatty plaques in the artery walls. One probable mechanism is mediated by the so-called foam cells described by the uncontrolled uptake of cholesterol and other lipids by macrophages which is presumably triggered by oxidized lipids [167].

The first hint to the involvement of chlorinated lipid species in atherosclerosis was obtained in 1996 [168]: Hazen and coworkers detected different chlorinated lipids upon the exposition of human LDL to the complete MPO system at acidic conditions with the focus on cholesterol: beside dichlorinated cholesterol, cholesterol *α*-chlorohydrin (6*β*-chlorocholestane-(3*β*,5*α*)-diol), cholesterol *β*-chlorohydrin (5*α*-chlorocholestane-(3*β*, 6*β*)-diol), and a structurally related cholesterol chlorohydrin could be detected and characterized by MS. The authors provided additional evidence that Cl_2_ derived from HOCl (particularly at acidic conditions which favor the presence of the free acid HOCl) is the actual chlorinating intermediate in the oxidation of cholesterol by MPO. It was suggested that Cl_2_ generation in acidic compartments constitutes one important pathway for the oxidation of LDL cholesterol in the artery wall.

A few years later, the presence of lipid chlorohydrins could be unequivocally confirmed in atherosclerotic lesions and it was also found that chlorinated lipids exhibit serious effects on different cell lines which may contribute to the development of the disease. These findings suggest that PL chlorohydrins formed in HOCl-treated LDL could contribute to the proinflammatory effects observed for this modified lipoprotein in vitro [169]. An interesting experiment was performed by Yang and coworkers in 2006 [170]: they infused clamped carotid arteries with 1 mM HOCl for 1 h, before reperfusion and animal recovery for up to 2 weeks. Interestingly, the results of these studies indicate that the addition of HOCl alone can lead to neointima formation containing both VSMCs (vascular smooth muscle cell) and macrophage infiltration that is consistent with the process of atherosclerosis.

A surprising observation was made in 2008 [171]. Although it is commonly accepted that the LPC levels are elevated in sera from patients with atherosclerosis and in atherosclerotic tissues [172], these are regularly saturated LPC species, as the majority of biologically relevant PLs contain an unsaturated fatty acyl residue in the* sn*-2 position and LPCs are typically generated by the enzyme PLA_2_ which cleaves the fatty acid in the* sn*-2 position. The authors demonstrated for the first time that LPC-chlorohydrins are elevated over 60-fold in human atherosclerotic lesions and therefore may have unique proatherogenic properties compared to common saturated LPCs. This emphasizes the role of plasmalogens in the disease [110]. The important role of plasmalogens in AP could already be shown in 2003 by Thukkani and coworkers as they found a 1400-fold higher concentration of the *α*-chloro fatty aldehyde (*α*-ClFALD) 2-ClHDA compared to normal aorta samples [173]. Interested readers will find more information about the role of plasmalogens in atherosclerosis in a recent review by Ford [174]. It has also been hypothesized recently that the type of the generated LPC is of (patho-)physiological relevance [175], because LPC is a major constituent of oxidatively modified LDL and is generally considered to be atherogenic. However, some studies have also shown antiatherogenic properties of some LPC species [175]. These controversial findings are apparently caused by changes of the degree of saturation of the fatty acyl moiety of the LPC species. The presence of (*ω*)-PUFAs at the* sn*-1 position of LPC makes LPC anti-atherogenic [176], while the presence of saturated fatty acids renders LPC atherogenic [177]. It is important to note that LPC is not only generated by PLA_2_, but LPC may be considered as a general product of the decay of oxidized lipids. This aspect will be discussed in more detail below.

### 7.2. Arthritis

Upon acute inflammation, the synovial fluid from patients suffering from rheumatic diseases usually contains a large number of granulocytes and cell numbers of the order of about 1 × 10^9^ cells in 30 mL synovial fluid are not exceptional [178, 179]. Since neutrophils (beside macrophages) are a rich source of the enzyme MPO, it is not surprising that synovial fluids are also characterized by significant MPO activities [120]. It is well known from investigations using classical assays such as the determination of thiobarbituric acid reactive substances, lipid hydroperoxides, or diene conjugates that the lipid compositions of synovial fluids change significantly under inflammatory conditions [180]. Although little attempts were performed to characterize the entire profile of oxidized lipids in the synovial fluids [181], it could be shown in 2005 that the LPC content correlates with the severity (inflammatory state) of the disease and even the success of a medical cure could be monitored by the decreasing LPC content [182]. In 2015 novel peptidoaldehydes from GSH and *α*-ClFALD in human neutrophils and a mouse model could be identified for the first time, indicating that *α*-ClFALD is produced as a result of MPO activity [183]. There is also increasing evidence that LPC represents just a transient compound [184]: lysophosphatidic acid (LPA) is mainly produced by the hydrolysis of LPC catalyzed by the enzyme lysophospholipase D, which is also called autotaxin (ATX). LPA interacts with specific G-protein coupled receptors and is involved in the regulation of cellular survival, proliferation, differentiation, and motility. Therefore, an elevated LPC content (mediated either by ROS or PLA_2_ activity) may be considered an important prerequisite of LPA generation. The inhibition of the LPA receptor has also been suggested as a promising way to suppress the symptoms of arthritis [185].

### 7.3. Other Diseases

There are many other diseases, for instance, multiple sclerosis, pulmonary fibrosis, liver fibrosis, and hepatitis, which are characterized by elevated LPC contents and/or enhanced MPO activities and/or enhanced lipid peroxidation product contents. These diseases have recently been reviewed and the interested reader is advised to consult [186].

## 8. Analysis of Chlorinated Lipids Using Different Mass Spectrometry Methods

Lipid oxidation products are classically assessed by photometric assays such as the determination of the thiobarbituric acid reactive substances (TBARS), the diene conjugates, or the number of hydroperoxides. Although these assays are simple and sensitive [187], they are not the methods of choice to determine chlorinated lipids. MS techniques using different ionization methods are much better suited for that purpose and will therefore be shortly discussed in this chapter. The HOCl-induced oxidation of plasmalogens was also investigated by MS, where plasmalogens gave even higher yields of LPC than diacyl-PCs with the same number of double bonds [109], confirming the extreme oxidation-sensitivity of plasmalogens. Since the analysis of FFA is easier in comparison to (phospho)lipids, they were formerly nearly exclusively investigated focusing on gas chromatography mass spectrometry (GC-MS) methods.

### 8.1. Gas Chromatography Mass Spectrometry

By using GC combined with different detection methods (i.e., ultraviolet (UV), MS, flame ionization detector (FID), or fluorescence) FFA oxidation products can be easily studied and the identification of many different products is made possible. Although GC offers an excellent resolution (even the separation of isomeric FFA is possible), only volatile organic compounds are detectable, which requires a mandatory esterification step of the FFA (often the fatty acid methyl ester, FAME) [188]. For more detailed information about sample preparation and GC-MS procedure regarding FFA analysis see also [189]. Using GC-MS, increased concentrations of fatty acid chlorohydrins were identified in adipose tissue, which emphasizes the systemic inflammatory response in acute inflammation [162]. Nevertheless, it has to be stressed that even the oxidation chemistry of simple compounds is very complex and increases with the complexity (number of double bonds) of the compound of interest. For instance, even the oxidation of isolated linoleic acid yields dozens of different products and this sheds light on the complexity of the product pattern [190].

Since PLs are nonvolatile, their direct separation and analysis with GC-MS is not possible. Therefore, the analysis of the fatty acyl composition of PL species requires a time-consuming sample preparation including the PL hydrolysis into diacylglycerols and FFA followed by their conversion into FAMEs [188]. However, there are some applications for the analysis of oxidized and chlorinated lipids using GC-MS. Due to their considerable reactivity with HOCl, plasmalogens readily generate oxidation products (i.e., *α*-ClFALD) and LPLs that are suitable for GC-MS analysis. *α*-ClFALDs are both hard to ionize and relatively unstable under ESI conditions. Thus, the conversion with pentafluorobenzyl (PFB) hydroxylamine into oximes improves the detection sensitivity by GC-MS, which is better in comparison to the achievable sensitivity of ESI MS [191]. In this study 2-chlorohexadecanal was investigated by GC negative ion chemical ionization (NICI) MS after PFB hydroxylamine derivatization of tissue and cell culture samples as an alternative method to LC-MS.

### 8.2. Electron Ionization Mass Spectrometry

Significant progress could be made in the last decades regarding the MS analysis of nonvolatile and/or high mass products. Until the eighties of the last century conventional electron ionization (EI) MS was nearly exclusively available. Using this approach, the sample is evaporated and subsequently ionized in the gas phase by collision with accelerated electrons, which (normally) leads to the removal of one electron from the analyte and the generation of radical cations. The most important prerequisite is sufficient volatility of the analyte. Therefore, this method can hardly be applied to lipids but is applicable to fatty acids subsequent to their conversion into methyl or trimethylsilyl esters to enhance their volatility [192].

Although the application range of EI is limited, the need to use volatile compounds enables the direct and straightforward combination of EI MS with GC. Among all chromatographic methods, GC offers the best separation quality for FFA and enables even the separation of different isomers. For instance, Winterbourn and coworkers [193] were able to show that both 9,10-chlorohydrin isomers of oleic acid can be differentiated by GC and that both isomers are generated in the same amount if oleic acid reacts with HOCl. Therefore, it can be concluded that the reaction between HOCl and unsaturated fatty acids is not regiospecific at all but results in a mixture of isomers. These authors have also suggested that the incorporation of chlorohydrins into cellular membranes leads to the destabilization of the lipid membranes, chlorohydrins, thus representing potential biomarkers. Similar data could be obtained by the same group regarding the chlorohydrins of cholesterol [194]. However, the biological significance of lipid chlorohydrins so far remains to be elucidated in more detail and there is also increasing evidence that the reaction between FFAs such as oleic acid and HOCl is even more complex than initially assumed. Recently, Schröter and colleagues [103] found that in addition to chlorohydrin as the main product of the reaction between HOCl and oleic acid dimeric and trimeric products are generated, the abundance decreasing from dimer to trimer. These oligomeric products were monitored by using high performance TLC (HPTLC), electrospray ionization (ESI), and matrix-assisted laser desorption and ionization (MALDI) MS. These oligomeric products might be derived either from the contribution of free radicals or from estolide formation [195].

### 8.3. Soft Ionization MS

Since the 1980s, a continuous stream of very creative papers was published in which new “soft ionization” techniques were presented enabling the MS analysis of native and oxidatively modified lipids without the need of previous saponification and derivatization. In contrast to EI, soft ionization MS enables direct detection of the molecular ion of the analyte. Typical soft ionization methods are ESI, APCI, and MALDI that enable direct analysis of native lipids and the corresponding oxidation products without the need of sample derivatization (like in GC-MS methods). In contrast to EI, the sample is not ionized by the loss of an electron but by the addition of a cation (often H^+^ or Na^+^, which are both omnipresent in biological samples) or deprotonation. Radical cations are normally not observed.

Basically, ESI and APCI are “softer” methods and can also easily be coupled to chromatographic separation methods such as High Performance Liquid Chromatography (HPLC). By contrast, MALDI is the more convenient method which tolerates a significant extent of impurities. Also, it generates almost exclusively singly charged ions, which makes MALDI spectra interpretation much easier [196].

#### 8.3.1. MALDI-TOF MS

**Figure 9:**
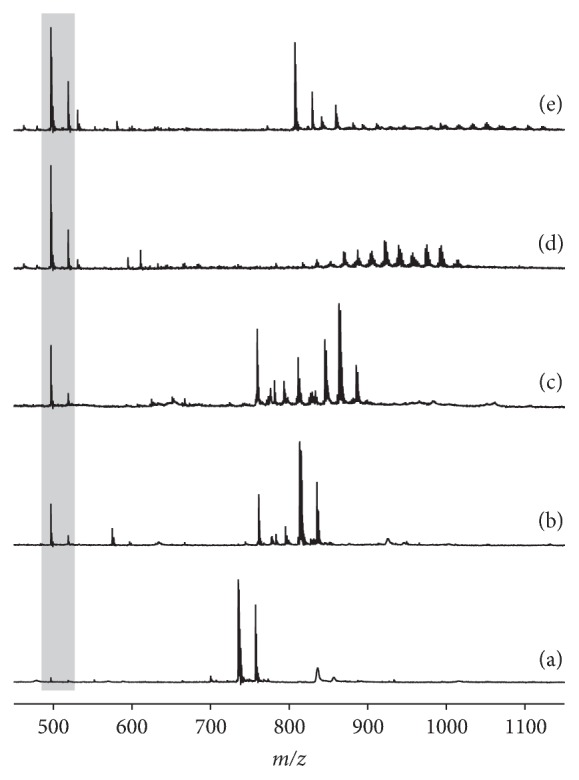
Formation of LPLs and higher molecular weight products (particularly chlorohydrins) after incubation of different PCs with an excess of HOCl as evidenced by positive ion matrix-assisted laser desorption and ionization-time of flight mass spectrometry (MALDI-TOF MS): (a) PC 16 : 0/16 : 0; (b) PC 16 : 0/18 : 1; (c) PC 16 : 0/18 : 2; (d) PC 16 : 0/20 : 4; (e) PC 16 : 0/22 : 6. Please note that the yield of lysophosphatidylcholine (LPC, marked by the grey box) increases with the extent of unsaturation of the used PC (reprinted with slight modifications from Arnhold et al. [198] with permission from Elsevier).

As early as in 2001, Arnhold and coworkers [197] were able to show that MALDI-TOF MS is a convenient method to study the effect of the reagent HOCl as well as the complete MPO/H_2_O_2_/Cl^−^ system on selected PCs with differently unsaturated fatty acyl residues. It could be shown by using polyunsaturated lipids (with arachidonoyl or docosahexaenoyl residues) that chlorohydrins and glycols are the most relevant products. The corresponding yields of these products could be influenced by varying the incubation time: monochlorohydrins and glycols dominated at short incubation times, while bischlorohydrins as well as products containing one chlorohydrin and one glycol moiety appeared after longer incubation. There was also evidence that LPC (lacking one fatty acyl residue in comparison to the native PL) is another important reaction product. This aspect was investigated one year later in more detail [198]. It turned out that the yield of LPC increases if PCs with a higher content of double bonds are oxidized by HOCl. This is due to the fact that PCs including an oleoyl residue (18 : 1) give only a small amount of LPC, while PLs with a docosahexaenoyl residue (22 : 6) result in considerable amounts of LPC. The generation of LPC under the influence of HOCl was explained by the introduction of electronegative elements (chlorine and oxygen) that withdraw electrons from the ester bond, thus enhancing its sensitivity to hydrolysis [198]. This is exemplarily shown in Figure 9. It is particularly notable that LPC may also be generated when phospholipases are completely absent.

Although this mechanism should be valid for all unsaturated lipids independent of the headgroup, the extent of LPL generation depends significantly on the structure of the used lipid: it was shown recently that the HOCl treatment of PE does not lead to LPE generation [199]. However, there is so far no convincing explanation for this surprising difference, and further studies to clarify this problem are urgently required.

In contrast to HOCl-induced lipid oxidations with LPC as the main product, hydroxyl radicals primarily cause scission at the position of the double bonds under generation of the corresponding aldehydes and/or carboxylic acids. This is a notable difference, although the detailed molecular reasons are still unknown. Finally, it has to be emphasized that a differentiation between HOCl and hydroxyl radicals under physiological conditions is still difficult: if an unsaturated PC is treated with the Fenton reagent in the presence of NaCl, the generation of chlorohydrins can be unequivocally monitored [200]. This implies that the in situ generated hydroxyl radicals react with the chloride under formation of HOCl. This might also be the reason why water radiolysis (the best characterized method of HO^∙^ generation) may also lead to the generation of HOCl if water radiation is performed in the presence of an elevated salt content [201]. This can be easily verified in cell experiment by measuring the number of dead cells in dependence of the salt concentration at a constant *γ*-dose [202].

Finally, a warning is required: although MALDI is a soft ionization method, it is less soft than ESI MS. While chloramines of PE can be easily detected by ESI MS [95], this is not possible by MALDI MS, at least if standard MALDI matrices but not the optimum matrix is used [203]: 4-chloro-*α*-cyanocinnamic acid is the MALDI matrix of choice for the detection of chloramines. Although not directly within the scope of this topic, the detection of peroxidized lipids by MALDI MS is also difficult. However, there are indications that their detection can be improved if the analytes are desorbed from a target coated with nitrocellulose [204].

#### 8.3.2. Native Lipid Mixture Analysis by ESI MS

As already indicated, ESI MS can be easily combined with chromatographic separation. Therefore, ESI coupled to HPLC (or more recently nano-HPLC) is normally the method of choice if complex lipid mixtures have to be analyzed [205]. This particularly applies because lipid oxidation products are normally only present in very small amounts in biological systems, while the native bulk lipids are by far more abundant [99]. Compounds which are present in small amounts only are normally suppressed by more abundant compounds and are thus often not detectable if the entire lipid mixtures are investigated without previous separation. The same applies if lipids with different headgroups and different tendencies to form a positive charge are investigated [206].

In a pioneering work Jerlich and coworkers [207] have shown that combined LC-ESI MS is a suitable method to monitor the oxidation products if PCs in human LDL are treated with either HOCl or the complete MPO system. It could be shown that chlorohydrin products from lipids containing oleic, linoleic, and arachidonic acids can be easily detected, but no hydroperoxides of linoleoyl or arachidonoyl lipids could be monitored. This is an important finding, since hydroperoxides were previously suspected to represent an additional reaction product which undergoes free radical-mediated reactions in the presence of HOCl [208].

Somewhat later the same authors [209] were able to show that there is a direct correlation between the content of oxidation products in the LDL sample on the one hand and the concentration of HOCl, or the MPO activity as well as the acidity of the medium, on the other hand. This could be also verified by the exposition of defined PC species to stimulated neutrophils [210]. It is nowadays commonly accepted that LC-MS is the method of choice to analyze lipid chlorohydrins, which has been reviewed recently [191]. Nevertheless, the limited commercial availability of oxidized PL standards aggravates the method development enormously. Compared to chlorinated cleavage products (*α*-ClFALD) of plasmalogens, their LPL chlorohydrins are easily detectable with ESI MS even without previous HPLC purification/separation [211]. These LPC-ClOH were detected by MS/MS using for example, the neutral loss scan (i.e.,* m/z* 95 for trimethylamine of the phosphorylcholine headgroup) [212].

#### 8.3.3. Native Lipid Mixture Analysis by APCI MS

Atmospheric pressure chemical ionization is a useful method which has facilitated lipid analysis for many years. Many classes of lipids including FFAs, PLs, sterols, and triacylglycerols are ionizable by APCI [213]. Although APCI is a soft ionization method, the relatively harsh conditions employed in the source induce some degree of fragmentation. This is sometimes helpful if no MS/MS capacity is available but aggravates the analysis of complex mixtures. Another problem regarding quantitative analysis is the strong impact of the extent of unsaturation on the ion yield. TAG can be conveniently analyzed by APCI, but APCI is not the method of choice to analyze PLs due to the relatively low sensitivity achievable. To the best of our knowledge there are only very few reports where APCI was used to analyze oxidized glycerolipids [214]. However, APCI is an excellent technique to analyze oxidized fatty acids [215]. Surprisingly, chlorinated lipids have so far not been analyzed by APCI.

### 8.4. Other Methods to Monitor Chlorohydrin Generation

There are not many methods [216] for evaluating the generation of lipid chlorohydrins apart from MS, but at least a few of them should be shortly mentioned.

#### 8.4.1. Nuclear Magnetic Resonance

Although Nuclear Magnetic Resonance (NMR) suffers from comparably low sensitivity, it has often been used to study the reaction between HOCl and unsaturated fatty acids or even PLs. ^1^H NMR was used in the majority of cases [217] since ^1^H is the most sensitive NMR nucleus. The progress of the reaction between the unsaturated lipid and HOCl can be easily monitored by the disappearance of olefinic residues and the formation of the typical chlorohydrin resonances that can be easily differentiated from the resonances of simultaneously generated glycols and epoxides [89]. Due to the limited sensitivity of ^13^C NMR there are no reports of ^13^C NMR studies of lipid chlorohydrins. However, there were a few attempts to use ^31^P NMR to monitor lipid oxidation products. Unfortunately, chlorohydrin generation cannot be easily monitored but is only reflected by a broadened resonance of the original PL with limited information [218]. By contrast, PC and LPC can be easily differentiated by ^31^P NMR, and even the position of the fatty acyl residue in the LPC species can be resolved.

#### 8.4.2. Spectroscopic Methods

Although infrared (IR) spectroscopy is surely suitable for the detection of N-Cl vibration bands which would be expected if PE or PS reacted with HOCl, there have so far been no attempts to study chloramines by IR. However, it could be shown that the reaction between for example, taurine, and HOCl is accompanied by the formation of an intense IR band at about 975 cm^−1^ [219], which is characteristic of N-Cl. This band is characteristic enough to confirm the presence of chlorinated compounds.

The standard method for following the reaction between HOCl and amines is UV spectroscopy, because mono- and dichloramines are characterized by a (weak) absorption in the UV range at about 254 nm [220]. Nevertheless, there is a problem if amphiphilic compounds such as lipids are investigated: although UV spectroscopy can be easily used in the case of water soluble compounds, for instance, phosphorylethanolamine or glycero-phosphorylethanolamine [65], the reliability of this assay is limited when PEs are investigated due to the light scattering effects caused by the formation of aggregates in an aqueous environment. This requires the application of suitable detergents to minimize unwanted aggregation.

#### 8.4.3. Chromatographic Methods

As already outlined (vide supra) HPLC is widely used to separate lipid oxidation products. Although TLC seems a bit old-fashioned in comparison to HPLC, it is also widely used in lipid research, as it offers many advantages, is inexpensive, and can also be performed by nonexperts [221]. Basically, the same separations performed by HPLC may also be accomplished by TLC. For instance, the effect of HOCl on cholesterol could be monitored by TLC [194] using normal phase TLC and diethyl ether, petroleum ether, and acetic acid (70/30/1, v/v/v) as eluent, while four different reaction products (the *α*- and *β*-chlorohydrins as well as the corresponding epoxides) could be monitored in addition to cholesterol. This also applies for more complex mixtures. Oxidation products of membranes from red blood cells could be detected by TLC although the authors were not satisfied with the TLC sensitivity and therefore also made use of an ELISA approach, which is more sensitive and could be successfully applied as soon as 1 × 10^6^ cells were available [153]. Nowadays, this sensitivity problem could also be overcome by combining TLC with MALDI MS detection, which provides a much higher sensitivity in comparison to TLC alone [222]. Applications of TLC have recently been reviewed in the context of plasmalogen oxidation product analysis [211].

#### 8.4.4. Immunological Methods

Such methods are widely applied to monitor the HOCl-induced modification of proteins by measuring, for instance, halogenated tyrosine residues [223] and, consequently, to determine the related MPO activities [224]. However, there are also antibodies available which recognize chlorinated fatty acids or lipids: in one of the first study an antibody against the chlorohydrin of oleic acid was developed [225], although this antibody was not very specific and also recognized some other chlorinated lipids. This method was also used to investigate the modification of red cell membrane lipids by HOCl [153], but otherwise this method is not frequently used.

## 9. Conclusions

Nowadays, it is commonly accepted that inflammation is accompanied by the generation of ROS and RNS. Since the majority of these reactive species are transient products and/or yield transient products as primary products, the establishment of reliable and specific “biomarkers” is still a challenging task.

The focus of this review was on the reaction between HOCl that is (i) used as an important disinfectant and (ii) generated under inflammatory conditions under catalysis of the enzyme MPO and different lipids. Nevertheless, the reactions of HOCl with some other biomolecules were also discussed, because they have functional groups which possess a higher reactivity than lipids. It was shown that lipids with exclusively olefinic residues (i.e., without reactive headgroups) yield mainly the corresponding chlorohydrins. Due to the stability of these reaction products, they may be easily characterized by various methods (particularly soft ionization mass spectrometry), and even the second order rate constants (*k*) of the reactions could be determined. Comparable results are obtained when HOCl is generated by the MPO/H_2_O_2_/Cl^−^ system mimicking physiologically relevant conditions. This reaction is important for two reasons: (i) Chlorohydrin formation may lead to a destabilization of the cellular membrane and (ii) lipid peroxidation may be initiated by the reagent HOCl. Additionally, it was shown that higher unsaturated lipids also yield LPC, even in the complete absence of phospholipases. This might be an explanation of the enhanced LPC contents in joint fluids in patients with rheumatoid arthritis, which are also characterized by elevated MPO activities. A variety of chlorinated lipids can be produced by the reagent HOCl or the complete MPO/H_2_O_2_/Cl^−^ system, but only *α*-chloro fatty aldehydes have so far been firmly demonstrated to occur during cardiovascular diseases, while evidence for other chlorinated lipids in disease and inflammation is still lacking. Since *α*-chloro fatty aldehydes can exclusively be generated by plasmalogen oxidation, this emphasizes the important role of these alkenyl ether lipids and the interest plasmalogens is currently attracting.

Compared to the reactions of cholesterol and PCs, the reaction between HOCl and lipids with reactive amino residues (e.g., PE and PS) has been by far less frequently investigated, although these lipids are also very abundant in biological membranes. This might be caused by the considerable product variability under these conditions, as the chloramines which are generated as the primary products are transient products and decay into aldehydes and nitriles but may also modify other functional residues of biomolecules.

Therefore, further attempts are necessary to study these reactions in more detail. This particularly applies as it was shown very recently that chlorinated products derived from PE and PS may impact the signal transduction pathways in cells.

## Figures and Tables

**Table 1:** Potential adverse effects in case of abuse or mishandling of industrial/medicinal used HOCl solution.

Field of application	Abuse/mishandling/adverse effects	Ref.
*Household products*		
Weakly concentrated solutions	Vomiting, pain, and dyspnea	[117]
Highly concentrated solutions	Nausea, diarrhea, hypotension, coma, hypernatraemic hyperchloraemic acidosis, convulsion, and cardiorespiratory arrest	[117]

*Water disinfection*	Oxidation of organic matter, anthropogenic contaminants, and iodide and bromide conversion in rivers, lakes, and groundwater	[118]

*Endodontics*	Chemical burns, tissue necrosis, neurological complications, and upper airway obstruction	[119]

**Table 2:** Survey of selected diseases where myeloperoxidase (MPO) presumably represents a suitable “biomarker.” The alterations of MPO concentration and/or activity are emphasized.

Disease	Changes of MPO	Matrix	Method	Ref.
*Arthritis*				

Rheumatoid arthritis	Increased activity/concentration	SF	ELISA	[120]
	Increased concentration	Serum	ELISA	[121]
		Plasma, SF	ELISA	[122]
	Increased activity	SF	NMR	[123]

Juvenile idiopathic arthritis	Increased concentration	Serum	Serum	[124]
		Plasma	ELISA	[125]

*Atherosclerosis*				

	Increased concentration	Plasma	ELISA	[126]
		Mixed with lipoproteins	ELISA	[127]
		AP	IHC; western blot	[128]
			HPLC, western blot	[129]
	Increased release by culprit plaque samples	CP	ELISA	[130]
	Increased activity	AP	Spectrophotometry	[131]

*Diabetes*				

	Increased level	Serum	Spectrophotometry	[132]
	Increased activity at higher G allele, anti-inflammatory effect of mutant A allele	MPO -463 G/A SNP	PCR	[133]
	Increased concentration	Plasma	ELISA	[134]
	Decreased activity	Neutrophilic cell lysate	Spectrophotometry	[135]

*Neurodegenerative diseases*				

Alzheimer's disease	Increased concentration	Plasma	ELISA	[136]
	Expression	Astrocytes	Confocal microscopy, IHC	[137]
	Increased Expression	Brain tissue	Immunoblot, IHC, PCR	[138]

Parkinson's disease	Expression	Brain tissue	IHC	[139]

Multiple sclerosis	Increased activity/concentration	Cerebral Cortex	Colorimetric analysis	[140]
	Expression	Microglia	IHC	[141]

*Systemic lupus erythematosus*				

	Increased concentration	Serum	ELISA	[121]
		Plasma	ELISA	[142]

*Lung diseases*				

Acute pulmonary tuberculosis	Increased concentration	Serum	EIA	[143]

COPD	Increased levels	Sputum	Meta-analysis	[144]
			ELISA	[145, 146]

Cystic fibrosis	Increased concentration	Sputum	ELISA	[147]

SF, synovial fluid; AP, atherosclerotic plaque; COPD, chronic obstructive pulmonary disease; CP, culprit plaque; EIA, Enzyme Immunoassay; ELISA, Enzyme-Linked Immunosorbent Assay; HPLC, High Performance Liquid Chromatography; IHC, immunohistochemistry; NMR, Nuclear Magnetic Resonance; SNP, Single Nucleotide Polymorphism.

## List of Abbreviations

AACL: Amino acid chloramine
AP: Atherosclerotic plaque
ATP: Adenosine triphosphate
ATX: Autotaxin
*α*-ClFALD: *α*-Chloro fatty aldehyde
COPD: Chronic obstructive pulmonary disease
CP: Culprit plaque
EI: Electron ionization (electron impact)
EIA: Enzyme Immunoassay
ELISA: Enzyme-Linked Immunosorbent Assay
ESI: Electrospray ionization
EPR: Electron paramagnetic resonance
FFA: Free fatty acid
FID: Flame ionization detector
GAG: Glycosaminoglycan
GC: Gas chromatography
HOBr: Hypobromous acid
HOCl: Hypochlorous acid
HOI: Hypoiodous acid
HOSCN: Hypothiocyanite
HNE: 4-Hydroxy-2-nonenal
HPLC: High Performance Liquid Chromatography
HUVEC-ST: Immortalized human umbilical vein endothelial cells
IHC: Immunohistochemistry
IR: Infrared
*k*: Second order rate constant
LC: Liquid chromatography
LDL: Low-density lipoprotein
LPA: Lysophosphatidic acid
LPC: Lysophosphatidylcholine
LPL: Lysophospholipid
MALDI: Matrix-assisted laser desorption and ionization
MPO: Myeloperoxidase
MS: Mass spectrometry
*m/z*: Mass over charge
NADPH: Nicotinamide adenine dinucleotide phosphate
NET: Neutrophil extracellular trap
NICI: Negative ion chemical ionization
NMR: Nuclear magnetic resonance
NaOCl: Sodium hypochlorite
Nrf2: Nuclear factor erythroid 2-related factor 2
oxPAPC: Oxidized 1-palmitoyl-2-arachidonoyl-*sn*-phosphatidylcholine
PA: Phosphatidic acid
PC: Phosphatidylcholine
PE: Phosphatidylethanolamine
PFB: Pentafluorobenzyl
PL: Phospholipid
PLA_1_: Phospholipase A_1_
PLA_2_: Phospholipase A_2_
PMNs: Polymorphonuclear leukocytes
PPARs: Peroxisome proliferator-activated receptors
PS: Phosphatidylserine
PUFA: Polyunsaturated fatty acid
RCS: Reactive chlorine species
RNS: Reactive nitrogen species
ROS: Reactive oxygen species
SF: Synovial fluid
SNP: Single Nucleotide Polymorphism
SOD: Superoxide dismutase
TBARS: Thiobarbituric acid reactive substances
(HP)TLC: (High performance) thin-layer chromatography
TMB: 3,3′,5,5′-Tetramethylbenzidine
TNB: 5-Thio-2-nitrobenzoic acid
TOF: Time-of-flight
UV: Ultraviolet
VSMC: Vascular smooth muscle cell.
